# Perspectives and Experts of Human Resource Management in Nursing During Disasters and Emergencies: A Qualitative Content Analysis

**DOI:** 10.1155/nrp/9921673

**Published:** 2025-10-07

**Authors:** Mahmood Nekoei Moghadam, Halimeh Kamali, Mohammadreza Amiresmaili, Seyed Mobin Moradi

**Affiliations:** Health in Disasters and Emergencies Research Center, Institute for Futures Studies in Health, Kerman University of Medical Sciences, Kerman, Iran

**Keywords:** disaster, emergencies, human resources, management, nurse

## Abstract

**Background:**

Disasters create an environment characterized by chaos and uncertainty, often leaving nurses feeling unsupported by their management. Despite this, nurses play a crucial role in the healthcare system's response during such crises. Therefore, this study aims to explore the perspectives of experts on human resource management in nursing during disasters and emergencies.

**Methods:**

This study employed a conventional qualitative content analysis design. Purposive sampling was used to recruit 21 experts and disaster managers in Iran between February and April 2025. Data were collected through semistructured interviews, which were audio recorded with participants' consent, transcribed verbatim into Word documents, and imported into MAXQDA software (Version 2020) for systematic organization and analysis. The data were analyzed using Graneheim and Lundman's (2020) qualitative content analysis method.

**Results:**

The analysis of the findings resulted in one main theme, poor nursing human resource management in disasters, and five subthemes, ineffective command process, passive presence and withdrawal, ineffective employment of volunteer nursing, insufficient attention to safety, and insufficient reinforcement of motivation.

**Conclusion:**

Given our ongoing observations of nurses' capabilities during disasters and emergencies, it is expected that the results of this study will aid planners, policymakers, and health sector managers in effectively allocating nursing manpower in potential future disasters. This will improve the quality of services offered to injured individuals and their families during such events.

## 1. Introduction

Disasters and emergencies can happen anytime. They often harm society's ability to meet health needs and provide care. Depending on their severity and significance, they can also lead to injuries, as well as financial and human losses [1]. Thus, aiding those injured in disasters is crucial, and the health system plays a vital role in assisting the wounded [1]. Disasters, depending on their scope and nature, increase the demand for essential services, which can overwhelm the operational and safety capacity of the healthcare system [2]. The system's usual functioning is disrupted compared to normal conditions [3]. Additionally, the effects of socioeconomic, technological, and cultural changes are pushing the health system toward greater complexity [2]. A key factor for ongoing success and achieving goals in these complex conditions is having qualified human resources [2].

Human resources are key to organizations. They need attention to build strong healthcare systems during crises [4]. In emergencies, high demand and few healthcare workers make effective management policies essential. These policies help sustain the workforce needed to manage disasters and provide care [5]. Good human resource management improves healthcare quality, optimizes resources, and reduces staff workload [6]. Health services rely on human resources, but global healthcare systems face challenges like shortages and uneven skills. Using effective management practices can boost skills, enhance service quality, and address these issues [5].

Despite the significant disruptions that disasters can cause in the health sector, organizations must still deliver quality services. Ensuring that healthcare facilities remain operational during disasters requires foresight, strategic planning, and diligent effort. When a disaster strikes, organizations face various challenges related to human resource management. For instance, the COVID-19 pandemic has not only tested existing systems and processes but also challenged the assumptions that underpinned their development [7]. This pandemic has presented unprecedented challenges to healthcare organizations and has underscored the critical importance of human resource management, particularly the role of nurses. Therefore, it is essential to develop human resource management plans and policies that ensure continuity of operations during disasters [7].

Qualified healthcare professionals are considered a fundamental component of the health system's response during emergencies [8]. Nurses, who represent the largest group of healthcare professionals, have a substantial history of performing their duties in response to both natural and human-induced disasters [9]. Their effective services are always in demand, as they are crucial members of the health service system during crises [10]. However, the role of nurses in the disaster response was not formally recognized until the publication of the International Council of Nurses (ICN) Disaster Nursing Competencies Framework in 2009 [11, 12]. Nurses are typically among the first responders at the scene following a disaster. Consequently, they must utilize their skills and efficiency to provide care to the victims, thereby preventing the situation from worsening and avoiding potential complications [13]. As key healthcare providers, nurses face widespread disasters, and their knowledge and level of preparation significantly impact their effectiveness before, during, and after such events [14]. Despite their critical role, nurses often report feeling abandoned by management during disasters and emergencies, which creates an atmosphere of chaos and uncertainty [9]. Strong management is essential in disaster situations, where an increase in casualties and patients can overwhelm the nursing workforce's capacity to respond, disrupting acceptable patterns of care delivery.

The optimal management of human resources in the health sector is regarded as a critical responsibility of managers. This includes enhancing employees' competence, increasing their knowledge, and developing their skills [15]. The importance of effective human resource management in healthcare can be understood from the perspective of the World Health Organization (WHO). According to a report by the WHO, a significant portion of government resources is allocated to health sectors, with human resources being the most crucial aspect [16]. This evidence highlights the importance and significance of optimal human resource management for healthcare systems.

Over the last thirty years, the healthcare system has undergone significant changes, transforming the role of the nurse manager. The position has become more complex as new expectations and expanded responsibilities have been incorporated into the job description [17]. Nurse managers serve as a crucial link among patients, nursing staff, medical professionals, other clinical and support personnel, and hospital administration, overseeing both human and financial resources [18]. Additionally, the level of engagement of nurse managers has been recognized as a factor influencing organizational productivity and profitability [19].

In times of disaster, the preparedness and knowledge of managers in healthcare settings, along with their understanding of disaster management, enable them to strategize effectively and respond appropriately during crises. This allows them to fulfill their responsibilities as needed [20]. These managers must leverage their skills and make prompt decisions to provide the necessary care, thereby preventing further complications and worsening of issues [21].

In Iran, numerous research projects have focused on emergency and disaster management or examined the involvement of hospitals and medical teams during these events. However, the contributions of nurse managers, who have been instrumental during crises in Iran, have not been explored. Given that nursing managers play a crucial role in recognizing and managing critical situations, leveraging their experiences could aid in the prevention and handling of future emergencies and disasters in the country. Hatami and colleagues (2017), through a descriptive assessment and analysis of hospitals' preparedness for crises concerning both facilities and personnel, determined that although nurses play a vital role in the initial response to crises, this group lacks adequate preparation and resources to provide services during such emergencies [22]. Similarly, Rahmati and colleagues (2018) indicated, following a cross-sectional study conducted in the emergency department of Military Hospital, that personnel struggled with evaluating and prioritizing patients for quick release and monitoring their conditions during a crisis [23]. Therefore, greater attention should be given to the management of nurses to enhance the quality of their services, maintain their health, and improve their productivity during disasters. This study aimed to explore the perspectives of experts on nursing manpower in times of disasters. It is anticipated that the findings will assist nursing managers in addressing the challenges they face during emergencies and disasters.

## 2. Materials and Methods

### 2.1. Study Design

This study was conducted using a qualitative approach with the content analysis method. The choice of this approach was influenced by its ability to systematically analyze and interpret open-ended data [24], making it particularly valuable for understanding the insights of nursing and disaster managers. In qualitative content analysis, narrative data are examined to identify and characterize prominent themes and patterns within those themes [25]. This method is useful when the research question involves understanding the meaning, themes, or patterns in the data, as it provides a structured and repeatable way to identify and classify these elements [26]. This study adhered to the Consolidated Criteria for Reporting Qualitative Research (COREQ) standards for qualitative research reports [27].

### 2.2. Participants and Setting

This qualitative study utilized conventional content analysis to explore the perspectives and insights of experts on human resource management in nursing during disasters and emergencies in Iran. The research population included nursing and disaster managers with both operational and executive experience. The study employed a purposive sampling method. Inclusion criteria required participants to have experience in hospital disaster management, specialized knowledge of disasters, and nursing experience within the health system. Exclusion criteria included unwillingness to participate in the study, reluctance to share personal experiences, lack of managerial experience, and absence of disaster management experience. The sample comprised faculty members, disaster managers, disaster and emergency experts, hospital managers, hospital matrons, and hospital supervisors, among others. Eligible participants were required to have at least 5 years of work experience, including a minimum of 2 years in a management role, and must have consented to participate in the study. A total of 21 participants collaborated in the study from February to April 2025 (see Table 1).

### 2.3. Data Collection

Data were collected through in-depth semistructured interviews conducted individually. Before the interviews, the researcher established a professional and effective relationship with the participants by clarifying the research goals and outlining the interview procedure, thereby fostering their confidence and preparedness. The participants were well-informed about the researchers, including their objectives, the rationale behind the study, and the researchers' passion for the subject. Essential explanations were provided to build trust and encourage involvement. The researcher demonstrated impartiality and strong communication skills throughout the interviews. Eligible participants were consulted, and the date and location of the interviews were mutually agreed upon. The demographic data of the participants, including age, gender, education level, current position, and work experience in nursing or disaster management, were recorded. All interviews were recorded with the participant's knowledge and consent. The interviews began with open-ended questions and then progressed to more specific ones. The primary questions asked included, “What do you think about the state of nursing human resource management in disasters? How should it be?” and “If you have learned any lessons from a disaster or an emergency, could you please explain them?” Finally, participants were invited to share any additional comments regarding nursing manpower management in disasters and emergencies. Additionally, probing questions were used to seek further clarification or to elicit more information about the components of nursing human resource management in such situations. Examples of these probing questions are provided in Table 2.

Data collection was conducted with maximum diversity in terms of age, gender, and experience in hospital disaster management or crisis management. The data collection continued until saturation was reached, which occurred after conducting seventeen interviews. Data saturation is commonly used in qualitative research methods such as interviews, focus groups, content analysis, or ethnographic studies [28]. During the data analysis phase, researchers determine whether data saturation has been achieved by assessing if new findings offer unique insights or merely repeat previously gathered information. To confirm data saturation, four additional interviews were conducted. In this study, due to the clarity and transparency of the initial codes obtained from the interviews, repeated interviews with participants were not necessary. As a result, data collection concluded after a total of 21 interviews with 21 experts in nursing and disaster management. All interviews were conducted by the corresponding author, Mrs. H.K., MSN (Adult Critical Care Nursing), PhD candidate (Health in Disaster and Emergencies), who has substantial experience in conducting interviews. The corresponding author and other team members are experienced qualitative researchers with multiple published articles in various academic journals. The interviews took place in a quiet, private setting after working hours at the participants' workplaces and lasted between 40 and 70 min, with an average duration of 55 minutes.

### 2.4. Data Analysis

#### 2.4.1. Conventional Content Analysis

The qualitative content analysis proposed by Graneheim and Lundman was utilized in this study [29]. Interviews were digitally recorded, transcribed verbatim, and then reviewed, coded, and analyzed. Data analysis was conducted simultaneously and continuously alongside the data collection process. For initial coding, the researchers used participant statements and index codes derived from the interviews. Initial codes were extracted from the interviews as meaningful units of participant statements. These codes were reviewed multiple times and categorized based on their similarity and relevance. Subsequently, similar codes were merged, and the categories were further refined, leading to second-level coding. In the next step, the categories were compared with one another, and those with similar characteristics were combined to create broader categories. The codes obtained from the data analysis were continuously reviewed and revised until the final stages of writing the study. The initially extracted codes were reduced during ongoing data analysis and comparison, resulting in the finalization of the main concept and categories related to the perspectives of experts on human resource management in nursing during disasters. MAXQDA20 (Version 2020) software was employed to code and extract categories and themes.

#### 2.4.2. Trustworthiness

To establish the validity and reliability of data or quality control, the four criteria (dependability, credibility, confirmability, and transferability) proposed by Lincoln and Guba (1985) were used [29]. Additionally, member checking was employed alongside the extended engagement of the researcher to enhance the trustworthiness of the data. Furthermore, after the coding process, the interview transcripts were sent back to the participants to verify the accuracy of the codes and the associated interpretations.

The peer check approach was employed to ensure data confirmability; for this, the data underwent coding and categorization, which were subsequently reviewed by the research team. If there was a lack of agreement regarding the codes and categories, discussions would persist to clarify the matter and attain a consensus.

To ensure the dependability of the data, an audit trail was employed. In applying this technique, the researcher retains the initial data, categories, and themes throughout the entire research process. Additionally, having two qualitative researchers who are not part of the study review and analyze the data from experienced individuals enhanced the credibility of the study.

The transferability of the research also relied on the assessment and validation of the findings by individuals within the same context. Using a sampling method that maximized variance contributed to the stability and transferability of the results, along with enhancing the credibility of the data. Dedicating adequate time to the study and engaging in face-to-face interactions with participants were additional elements that bolstered the credibility of the data. The results were also validated by several managers who did not participate in the study.

### 2.5. Ethical Considerations

This study received approval from the Ethics Committee of Kerman University of Medical Sciences (IR.KMU.REC.1403.004), and the necessary permissions to conduct the research were granted. The research adhered to the ethical standards outlined in the Declaration of Helsinki. The researchers introduced themselves to the participants and thoroughly explained the objectives of the study and the methods of data collection. Written informed consent was obtained for both participation in the study and audio recording. The timing and location of the interviews were arranged according to the participants' preferences. Participants were assured of the confidentiality of their data throughout all phases of the research and were informed that they could withdraw from the study at any time. Furthermore, participants were invited to contact the researchers if they had any questions or concerns.

## 3. Results

The mean age of the participants was 48.6 years. Additionally, 57.1% of the participants were male, and most held postgraduate or postdoctoral degrees. The average work experience among the participants was 23.8 years. The participants were nine faculty members from universities of medical sciences, two experts from the Red Crescent Society of Iran, one expert from the Tehran Municipality Disaster Management Organization, two matrons, two clinical supervisors, one hospital manager, one nursing deputy from the Ministry of Health, and three disaster managers from universities of medical sciences.

Through the analysis of the interviews, the researchers identified a main theme: Poor management of nursing human resources in disasters and emergencies. The study also revealed five sub-themes or major challenges: Ineffective command process, Passive presence and withdrawal, Ineffective employment of volunteer nursing, Insufficient attention to safety, and Insufficient reinforcement of motivation (see Table 3). Each major challenge included minor challenges, which are explained below with participant quotations. Accordingly, essential solutions to address these challenges were proposed (see Table 3).

### 3.1. Sub-Theme 1: Ineffective Command Process

The absence of a unified command during disasters and emergencies is one of the most significant issues reported, resulting in numerous consequences. One participant stated, *“The main problem is the parallel work, and many procedures are repeated. That unified command structure does not exist” (P21).* Another participant noted, *“The issue arises when volunteers join the team for disaster management, leading to redundancy and the creation of overlapping tasks” (P4).*

Participants emphasized the necessity of establishing a unified incident command system for managing disasters and emergencies. One participant remarked, *“There is a need for a unified command cycle to manage nurses and other human resources” (P2).* They also highlighted the importance of quickly reviewing the roles and responsibilities defined by the incident command system: *“When a disaster occurs, an alarm should sound in the hospital. The hospital manager serves as the commander during emergencies. Furthermore, all responsibilities and roles should be reviewed promptly, and each person's position should be clearly defined” (P18).* Another participant added, *“When a crisis occurs, an alarm should sound throughout the hospital. We have a crisis flowchart, and in this flowchart, the chief and disaster commander is the hospital director” (P5).* However, as Participant 3 pointed out, disaster and emergency management charts are often not effectively utilized during crises: *“Unfortunately, hospitals have red and yellow charts… but they don't know what to do in times of crisis practically” (P3).*

### 3.2. Sub-Theme 2: Passive Presence and Withdrawal

Most participants believed that some volunteers might become emotionally involved in the operation and may not consistently cooperate with the crisis team due to their unique behavioral and psychological characteristics, potentially leaving the team before the response process is complete. One participant noted, *“Some volunteers may be excited at first and express themselves in the field, but they may not cooperate until the end and leave the team” (P20).*

Participants expressed concern that during a disaster, some nursing staff might leave the operation for various reasons, rendering them unable to provide services. During such times, some staff may experience physical and mental injuries or trauma during the response, or they may be unable to continue working due to underlying illnesses or their recurrence, as well as physical and mental vulnerabilities. *“A volunteer nurse may leave the team due to psychological stress” (P10).*

Some participants also pointed out the lack of cooperation and full participation among younger nursing staff, along with the need for evidence-based practices related to disease management. They shared their recent experiences during the COVID-19 pandemic: *“The story was that the staff who were new to the job wanted to be less involved in the hospital during the crisis, and they were constantly bringing papers from different doctors stating that they had COVID-19” (P12).*

Most participants believed that the challenges mentioned would lead to a shortage of nursing staff. Although the issue of nursing staff shortages is common in Iran, even under normal conditions, they suggested developing the capacity of nursing staff by issuing a call to address these identified challenges. Participants mentioned that this call could be disseminated through medical universities and mass media. Participant 13 stated, *“Our calls are only on paper, and I would face problems if a crisis occurred. It would be better if it were done systematically through medical universities and mass media” (P13).*

To increase capacity, the potential of retired nurses, nursing students, and other disciplines at the University of Medical Sciences could be utilized, along with clinical and nonclinical supervisors and nurses working in clinics, nontherapeutic units, and administrative departments. Participants also mentioned the temporary extension of planned nursing workforce initiatives. *“In my opinion, one solution for managing nursing human resources during a crisis is to utilize volunteer workers. These volunteers could be nurses who are currently unemployed, retired nurses, or even nursing students who are completing internships. They can serve as effective volunteers” (P6).*

One way to address the shortage of nursing staff and increase nursing capacity during a crisis is to recruit temporary or short-term nursing staff. *“We can train temporary nursing staff through short-term courses lasting three to six months; they can provide significant assistance” (P1).* The 89-day nurse recruitment plan implemented during the COVID-19 pandemic was referenced: *“When they recruited nursing staff for 89 days, it opened doors for many to come to work and give their best” (P2).*

Some participants believed that during times of crisis, the capacity of professional volunteers from supporting organizations could be utilized by issuing a call and providing short-term training in relief or nursing assistance; these individuals could serve as temporary nursing staff during emergencies. Participant 10 reflected on his experience during Iran's past imposed war: *“Additionally, we should create the capacity to use volunteers. During the war, when we lacked enough doctors or nurses, we were able to meet some needs with short-term relief courses that lasted from 15 to 45 days” (P10).*

Some participants noted that in the event of an internal crisis and a shortage of nursing staff, paid leave is typically canceled in hospitals, and nurses are often offered voluntary overtime. *“Sometimes the leave is canceled; we try to reduce the time off they want to take and encourage them to work more overtime” (P5).*

### 3.3. Sub-Theme 3: Ineffective Employment of Volunteer Nursing

The participants emphasized that the competencies of volunteers from supporting organizations are not thoroughly assessed. “*They join the field and participate in disaster response simply by applying, without any competency evaluation” (P17)*. Additionally, a majority of the participants in this study explained that the most significant challenge was the failure to employ nursing staff based on experience and competencies. Participant 20 recounted his experience during the earthquake as follows: *“I was a fourth-semester nursing student in City A. When the earthquake struck at midnight, we were instructed to go to the hospital for assistance. At that time, it was 1969, and there was no clear understanding of disaster response. I arrived at the hospital and saw a man applying a plaster cast. He noticed my gown and asked, ‘What are you doing?' I replied that I was a nursing student and said, ‘Come on, I'll put on a plaster cast.' I'm sure several cases of compartment syndrome occurred that night” (P20)*. *“*For example*, during the COVID-19 outbreak, students worked in the ICU for COVID-19 patients, and we observed an increase in the death rate in the ICU” (P13).* This situation not only exacerbates the workload of the disaster management team members but also exposes them to numerous challenges. *“The disaster manager must oversee volunteers who join the team. As you mentioned, not only do they fail to assist us, but they also become a burden” (P6).*

Consequently, the participants believed that both specialist and nonspecialist nurses should be clearly identified. Nonspecialist nurses should be employed in stable conditions to perform basic and less invasive procedures, while specialist nurses, as critical care nurses, should be hired in critical and frontline situations to carry out invasive measures and protocols. *“These staff members should be organized and employed based on initial assessments and eligibility criteria” (P1).*

Furthermore, critical care nurses can serve as supervisors for nonspecialist nurses. Some participants also stressed that young volunteer nurses should work alongside experienced nurses during disaster responses to minimize negative consequences: *“I should also mention that a nurse must supervise and assist in transferring victims by volunteers because the victims are at risk of developing additional injuries” (P15).*

### 3.4. Sub-Theme 4: Insufficient Attention to Safety

The participants in this study believed that during disasters and emergencies, insufficient attention is consistently paid to the safety of nursing personnel. Maintaining the safety of these individuals depends on their access to appropriate and high-quality safety equipment, which is unfortunately often neglected. *“There was no attention given to maintaining the safety of nurses. With the same substandard and low-quality PPE, nurses were performing very heavy and stressful work” (P19).* Another identified challenge was the unfair distribution of personal protective equipment (PPE) during disasters. Participant 8 shared his experience from the recent COVID-19 pandemic: *“In the early days of COVID-19, if you remember, there were very few masks, sanitizers, and personal protective clothing available in hospitals. Some nurses had more access, while others had less” (P8).*

Most participants emphasized that PPE should be distributed consistently and fairly among nurses during disasters and emergencies. *“A nursing manager should consider both the quantity and quality of the equipment they intend to provide and ensure fairness in its distribution so that it can be allocated appropriately” (P16). “As a manager, the priority in this situation should be fairness. Justice must be considered in the distribution of personal protective equipment among the staff” (P20).*

During an emergency, PPE should be distributed based on the level of vulnerability or the severity of the hazard in the area. *“If a nuclear accident occurs, nurses who are near the accident site, such as those entering the hot zone, should receive significantly more equipment than those who are farther away from the incident site” (P9).*

### 3.5. Sub-Theme 5: Insufficient Reinforcement of Motivation

Participants claimed that one of the main challenges in this context was the lack of positive feedback for nursing staff, emphasizing that the selflessness of nursing volunteers is often overlooked and undervalued by managers and officials. As a result, volunteer nurses may lack sufficient motivation to participate in future crises. *“Many times, we see nurses who dedicate their lives to helping during major events, but they don't receive any positive feedback; thus, they become demotivated because people need to be appreciated for the good things they do” (P3).*

Participants stated that during a crisis, one factor that boosts the motivation of nursing staff is the support and empathy of disaster managers. *“I was at the hospital every day, encouraging the staff. I think this is very effective” (P19).* Some participants emphasized that instead of merely managing nurses, the focus should be on leading them. *“Instead of managing nurses during a disaster, they should be led. Time should be given to each nurse individually. This approach will help solve many problems” (P7). “Why do they say that leadership and management are different? Because a leader stands in front of everyone and cares for them, while a manager is behind and pushes everyone away” (P21).* “To effectively guide the nursing staff, we need a leader rather than a manager” (P4).

Some participants emphasized the importance of honest and fair communication between managers and nursing staff during disasters. *“The communication between the manager and the nurse must be honest and fair. The way a manager treats nurses is very important. If this is not the case, it will lead to staff turnover because nurses are knowledge workers, and losing them will harm the entire organization; they are not ordinary staff” (P18). “A manager should not make any distinctions among their staff” (P5).*

Participants noted the importance of appreciation and financial rewards to compensate for participation. *“These individuals should receive financial support during disasters” (P11).* Some participants also believed that if rewards and incentives are provided, the process should be transparent and equitable. *“It is important that the incentives are fair and that all individuals are recognized so they remain engaged in future events” (P12).*

Several participants claimed that moral encouragement, such as written letters of appreciation and certificates of recognition during disasters, strengthens employees' motivation to continue participating. *“Even a very small letter of appreciation can encourage these individuals” (P15).* Participants also mentioned the significance of positive feedback from patients and their family members, as well as from the families of nursing staff involved in the crisis. *“This individual should do at least something that encourages their family to acknowledge their efforts, saying how well they are performing. Apart from their humanitarian work, their presence in disasters has made a difference, which is beneficial for us” (P17).*

Several participants also stated that another factor that enhances motivation during disasters is the support and empathy of the public and benefactors. *“We should also pay attention to the support and empathy from the public and benefactors. They consistently stand by nurses during disasters, and this support fosters a sense of encouragement among nurses” (P5).*

## 4. Discussion

Based on the findings of this study, the main theme identified was the poor management of nursing human resources in disasters and emergencies. The five sub-themes identified were: Ineffective command process, Passive presence and withdrawal, Ineffective employment of volunteer nursing, Insufficient attention to safety, and Insufficient reinforcement of motivation. These sub-themes are discussed in this section.

Nurses constitute the largest segment of healthcare professionals and are essential to disaster prevention [30]. During the disaster response phase, key elements of disaster management are implemented within the healthcare facility: triage is conducted, the injured receive emergency care, and patient evacuation and relocation are coordinated with other medical centers [12]. Exhaustion associated with heavy workloads can prevent nurses from taking on more challenging responsibilities [31]. Goniewicz and Goniewicz stated that nurses are uncertain about their responsibilities in disaster situations, and that this uncertainty presents significant challenges to disaster management [29]. Therefore, it is essential to acknowledge the challenges of managing nursing personnel in disaster situations to foster specialization in disaster management.

The results of the study show that the lack of a unified command and management framework, along with disruptions in delivering vital care, are the main obstacles to managing nursing staff during disasters in Iran. It is crucial to coordinate many volunteer nurses as a unified team during disasters to minimize redundant efforts and eliminate repetitive procedures. Wang et al. pointed out that supervising nurses as they carry out critical tasks, particularly during a disaster or emergency, has emerged as a significant issue [32]. The results highlight the importance of the incident command system. Regrettably, in Iran's healthcare system and hospitals, this system is largely represented by charts and banners, lacking practical implementation during critical situations. During the disaster-response phase, the incident command system must be actively engaged, with clearly defined roles and responsibilities carried out within this operational framework. These findings align with the study by Yousefi et al. conducted in Iran, which concluded that, to strengthen the capabilities of nurses and hospital personnel in the face of disasters, hospital incident command systems (HICS) should be implemented, accreditation guidelines should be formulated, and these standards should be practiced in healthcare environments [31]. Ultimately, various studies in Iran have indicated that, unlike in other nations, despite disaster-response committees having been established at the central headquarters of universities, emergency services, and most hospitals, very few possess a structured disaster-response plan, the formation of specialized teams, organizational charts, and clearly defined job descriptions; consequently, a notable lack of preparedness is evident [33].

The study also highlights significant challenges related to the inadequate assessment of volunteer nurses' qualifications and the neglect of nursing staff attitudes based on their experience and skills. Research conducted following the Bam earthquake in Iran revealed that approximately 88% of individuals were dissatisfied with the lack of specialized personnel, including doctors and nurses, as well as the presence of untrained volunteer groups in the affected regions [34]. During disasters and emergencies, it is crucial to effectively hire nurses to deliver high-quality services that enhance the satisfaction of both patients and the injured. Previous research consistently emphasizes the importance of qualifications, scientific evaluations, and expert knowledge in nursing care. For example, Cheraghi et al. demonstrated that it is vital for managers to consider nurses' evaluations regarding patient understanding and responsibility, as effective patient care goals depend on the attainment of knowledge and skills [35].

A systematic review of nurses in Iran revealed that their knowledge of disasters and emergencies is insufficient, which can be attributed to the lack of effective training programs and courses in hospitals [31]. Ghanbari et al. found that nurses exhibited inadequate disaster preparedness and suggested the introduction of training programs to enhance their readiness for emergencies [36]. Additionally, Hajavi et al. indicated that other healthcare staff in hospitals, such as medical records personnel, may have limited knowledge, possibly due to insufficient planning and the failure to implement disaster preparedness policies in these facilities [37]. A study by Najafi and Hamzeh Pour aimed to determine the knowledge and attitudes of Red Crescent volunteers regarding chemical incidents and found that none of the volunteers possessed sufficient knowledge in the area of chemical incident management. Additionally, only 8.3% of the volunteers demonstrated a suitable attitude toward managing chemical incidents, and only 5.8% indicated that they were prepared to be deployed to crisis areas affected by chemical incidents [38]. Sajjadi et al. concluded that most nurses, as well as paramedics, are unprepared and unfamiliar with chemical, microbial, nuclear, and radioactive incidents. This lack of preparedness is evident not only in Iran but also in developed countries [39]. According to Farrokhzad et al.'s study, fear of the unknown, inexperience in facing new situations, mistakes due to limited and contradictory information, continuous stress, and insufficient professional skills are the most significant challenges faced by nurses in emergencies and disasters [40]. Therefore, training on how to handle these incidents and fostering a culture among relief and treatment teams, including emergency nurses, is of great importance. This preparation ensures that they are ready and willing to provide relief and respond effectively in operational environments when necessary [39]. Consequently, it is recommended that a thorough assessment of the scientific knowledge and practical skills of volunteer nurses be conducted before their deployment in disaster or emergency scenarios. Although Labrague and Hammad found that national nurses often possess sufficient knowledge and skills related to disaster responses, they remain unprepared for such situations despite the risk of disasters [41].

Another finding from this study indicated that nurses can successfully fulfill their care tasks. First, it is essential to ensure access to key agencies and equipment that protect health and maintain the quality of care. However, managers expressed concerns regarding the significant shortage and inadequate access to high-quality and standard PPE for nurses in critical situations. Similarly, Wang et al. identified a lack of PPE during the COVID-19 pandemic [42]. As noted by Wang et al., hospitals throughout China, particularly in Wuhan, experienced a critical deficiency in medical supplies, especially PPE, including medical gowns and N95 masks, during the pandemic [42]. Consequently, to address this issue, hospitals called for increased public and social assistance [42]. Philanthropists in Iran consistently support victims and the injured following disasters. During the recent pandemic, these benefactors aided hospitals and medical facilities by supplying PPE and worked to alleviate issues such as PPE shortages. Research has shown that the scarcity of PPE poses significant challenges for managers striving to ensure the safety of nurses during crises. Nevertheless, the healthcare system has prioritized patient safety, often at the expense of nursing staff [13, 14]. Therefore, managers need to prioritize the safety of both nurses and patients, both under normal circumstances and in times of crisis.

Nurses play a vital role in the healthcare system, and their effective services are crucial during critical situations [43, 44]. It is important to emphasize that demotivated and disengaged nurses can decrease the efficiency of the healthcare system and lead to long-term negative effects on public health [45]. Consistently recognizing the efforts and contributions of nurses is essential, especially during times of crisis. Furthermore, during the response phase, the healthcare team should acknowledge the hard work and service delivery of nurses.

This study emphasizes the importance of enhancing motivation and encouraging participation among nurses during crises. The findings indicate that fostering a spirit of volunteerism among nurses can be achieved through both financial and intrinsic incentives. Significant attention has also been given to ensuring that these incentives are provided and distributed fairly. However, one participant, who has made substantial contributions to nursing management in Iran, opposed the idea of financial compensation for volunteers in disaster situations, arguing that those who offer their services voluntarily should not expect monetary rewards. It is essential to protect the interests of nursing volunteers and respect their values during disasters and emergencies [47]. Additionally, Matsumoto noted that compensation significantly influences the willingness to remain in a position and affects the career development of nurses. Salary is an environmental factor, and it is necessary to remain competitive at all times [46].

Based on the other findings of this study, it has been suggested that voluntary nurses should also be provided with spiritual incentives, such as positive feedback for the nursing staff, support and empathy from managers, a focus on leadership alongside management, fair and honest communication between managers and nurses, the provision of certificates of appreciation, and support and empathy from the public and benefactors. Additionally, conveying positive feedback from patients and their family members, as well as from the families of the staff, is crucial. Such measures are expected to strengthen the motivation and spirit of participation among nursing volunteers. The study conducted by Zamanzadeh et al. indicates that employing uninspired and disinterested nurses can diminish the efficiency of the healthcare system and potentially lead to severe harm to patients' well-being [45]. However, the results of this study revealed that deficiencies in the communication skills of the disaster team leader posed an additional obstacle to enhancing voluntary motivation. According to Shieh et al., the leadership team must possess interpersonal communication skills, foster innovation, demonstrate effective leadership, and have the requisite capabilities to establish partnerships [48]. As stated by Aditya et al., implementing innovations aimed at enhancing motivation—which in turn alleviates mental and physical fatigue—should be considered [49]. The results indicated that actions such as providing rewards and expressing verbal appreciation were identified as supportive measures by managers, aligning with the cultural norms of Iran. Sihvola et al. affirmed that positive reinforcement can enhance nurses' self-confidence and promote their autonomy [50]. According to García-Martín et al., nurses and managers need to collaborate closely as a cohesive team during disasters. The effectiveness of team leadership and management skills is crucial for successful disaster risk management [51]. The research revealed that the leader of the disaster team faced significant challenges due to inadequate communication skills, which adversely affected the management of nursing human resources during disaster response. Norris et al. proposed that integrating electronic medical information and technology into disaster management could enhance communication and streamline workflows between managers and healthcare professionals [52].

Finally, it is important to recognize that the workforce within the health sector is considered a vital resource for delivering services. To mitigate the impact of a disaster, medical professionals, particularly nurses, must be equipped to respond promptly. However, managers encounter numerous challenges at every stage of disaster management. Therefore, a comprehensive understanding of the existing circumstances is essential for making informed decisions regarding the improvement and development of personnel management in nursing during disasters and emergencies.

## 5. Limitations

One significant drawback of qualitative research is its inherent subjectivity and the potential influence of researchers' biases on the results. In this study, the researchers aimed to identify their pre-existing beliefs, attitudes, and personal experiences, documenting these factors to mitigate their impact on data analysis and interpretation. Additionally, some potential participants were hesitant to take part in the study. To address this issue, the interviewer sought to encourage cooperation by clarifying the research objectives and ensuring the confidentiality and privacy of the individuals being interviewed.

Another limitation was the possibility that participants would be unavailable for the interview. To tackle this challenge, the aims and significance of the study were explained to the individuals, and an interview schedule was arranged based on their preferences.

Moreover, a review of existing literature highlighted an urgent need for investigations into the management of the nursing workforce during crises and emergencies. As a result, much of the research has focused on the indicators and attributes of nursing in disaster situations and their contributions to disaster management. This study explored the insights of managers and specialists in emergency and disaster management, along with the perspectives of nursing managers who have been involved in previous disasters, regarding the state of nursing human resource management in emergency and disaster responses, and proposed effective and practical solutions. Furthermore, this research was conducted within the context of Iranian culture, which may limit the applicability of its findings to other nations with different cultural or contextual backgrounds.

## 6. Conclusions

Overseeing nursing staff during a disaster or emergency is crucial. In these situations, thorough planning and efficient organization are essential to ensure that health and nursing services are provided to those affected. A key component of this management is the optimal and efficient allocation of human resources. Additionally, establishing effective communication channels can enhance coordination between different teams, while developing clear crisis management strategies can significantly improve the overall effectiveness of nursing teams in crises. It is also important to prioritize the mental and physical health of nurses during these challenging times. These strategies not only enhance the quality of service during emergencies but also strengthen public trust in the healthcare system. While we continue to witness the incredible skills of nurses in disasters and emergencies, the findings of this research are expected to assist planners, policymakers, and health sector leaders in efficiently deploying nursing personnel in anticipated future disasters. This will improve the quality of care provided to those affected by such incidents and their families.

For future studies, it is recommended to design and conduct a systematic review study focused on identifying the essential elements of nursing human resource management during disasters and emergencies. Additionally, a mixed-method study should be developed to formulate a theoretical model for nursing human resource management in the context of disasters and emergencies.

## Figures and Tables

**Table 1:** Characteristics of participants.

Participant	Gender	Current position	Experience (years)	Education/field of study
P1	F	Faculty member	28	PhD/Healthcare Services Management
P2	F	Faculty member	21	PhD/Health in Emergencies and Disasters
P3	M	Faculty member	32	Postdoctoral, PhD/Health in Emergencies and Disasters
P4	M	Experts in the Red Crescent Organization	8	PhD/Nursing
P5	M	Hospital Matron	27	Master/Nursing
P6	M	Faculty member	28	PhD/Health in Emergencies and Disasters
P7	M	Tehran Municipality Disaster Management Organization	16	General Practitioner/Medicine
P8	F	Supervisor	26	Master/Nursing
P9	M	Supervisor	28	Master/Nursing
P10	M	Nursing deputy from the Ministry of Health	29	PhD/Nursing management
P11	F	Faculty member	19	PhD/Health in Emergencies and Disasters
P12	F	Disaster managers from the University of Medical Sciences	25	MPH/Emergencies and Disasters
P13	F	Faculty member	26	PhD/Health in Emergencies and Disasters
P14	M	Experts in the Red Crescent Organization	23	PhD/Health in Emergencies and Disasters
P15	M	Disaster managers from the University of Medical Sciences	28	Master/Nursing
P16	F	Faculty member	27	PhD/Nursing
P17	F	Faculty member	18	PhD/Nursing
P18	M	Faculty member	21	PhD/Nursing
P19	F	Disaster managers from the University of Medical Sciences	17	Master/Nursing
P20	M	Hospital Matron	20	Master/Nursing
P21	M	Hospital manager	29	Master/Nursing

*Note:* F, female; M, male; MSc, Master of Science; PhD, Doctor of Philosophy.

**Table 2:** Interview questions.

General question	What do you think about the state of nursing human resource management in disasters? How should it be?
Middle question	If you have learned any lessons from a disaster or an emergency, could you please explain them? Can you elaborate on that? What does that mean?
Final question	Are there any additional keywords, ideas, or statements you believe were missed during the interview?

**Table 3:** The challenges and solutions of nursing human resource management in disasters and emergencies.

Main themes	Sub-themes	Codes	Solutions
Poor nursing human resource management in disasters	1. Ineffective command process	The lack of a unified command in disasters and emergencies Performing parallel tasks and disrupting the provision of effective care Weaknesses in the operational execution of the Incident Command System	1. Formation of the Incident and Disaster Management Committee 2. Activation of the unified Incident Command System 3. Operational implementation of the Incident Command System
	2. Passive presence and withdrawal	The emotional presence of the nursing volunteer force Insufficient cooperation or engagement from the young nursing workforce Documentation of a healthy nursing force based on physical illness or injury Withdrawal of vulnerable and physically disabled individuals	1. Nurses call program 2. Temporary recruitment of nursing staff on short-term contracts with job security 3. Recruitment and extension of the nursing staff plan 4. Call for nursing students 5. Call for inactive health worker volunteers 6. Cancellation of paid leave of official nursing staff
	3. Ineffective employment of volunteer nursing	Limited competency assessment Failure to employ the nursing staff based on experience and competencies Failure to integrate senior nurses and nurses with less experience	1. Assessing competency 2. Scientific and illogical allocation of nursing duties 3. Integrating senior nurses and nurses with less experience 4. Utilize motivated young nursing staff
	4. Insufficient attention to safety	Inadequate and poor-quality safety equipment Unfair distribution Nondistribution of PPE based on the level of vulnerability or the severity of hazards in the area	1. Access to suitable and quality PPE 2. The continuous and fair distribution of suitable and quality PPE 3. Rationing of PPE 4. Distribution of PPE based on the level of vulnerability or the severity of hazards in the area 5. Supplying required PPE by benefactors and supporting organizations 6. Effective supervision of the use of PPE 7. Enhancing the safety of the workplace with a specialized team 8. Provide safety and health packages
	5. Insufficient reinforcement of motivation	Lack of positive feedback to the nursing staff The failure to provide financial and spiritual incentives Providing nontransparent and unfair rewards	1. Giving positive feedback to the nursing staff 2. Support and empathy from managers 3. Focus on leadership alongside management 4. Fair and honest communication between managers and nurses 5. Appropriate and fair financial incentives 6. Providing certificates of appreciation and spiritual incentives 7. Support and empathy from the public and benefactors 8. Transferring positive feedback from the injured and their family members 9. Transferring positive feedback from the staff's families

Abbreviation: PPE, personal protective equipment.

## Data Availability

The data used to support the study's findings are available from the corresponding author upon request.
